# PIAS1 Alleviates Hepatic Ischemia-Reperfusion Injury in Mice through a Mechanism Involving NFATc1 SUMOylation

**DOI:** 10.1155/2022/4988539

**Published:** 2022-08-31

**Authors:** Jing Luo, Jiequn Li, Ting Li, Zhongqiang Zhang, Guangshun Chen, Qiang Li, Haizhi Qi, Zhongzhou Si

**Affiliations:** Department of Liver Transplantation, The Second Xiangya Hospital of Central South University, Changsha 410011, China

## Abstract

Recently, attentions have come to the alleviatory effect of protein inhibitor of activated STAT1 (PIAS1) in hepatic ischemia-reperfusion injury (HIRI), but the underlying molecular mechanistic actions remain largely unknown, which were illustrated in the present study. Microarray-based analysis predicted a possible regulatory mechanism involving the PIAS1/NFATc1/HDAC1/IRF-1/p38 MAPK signaling axis in HIRI. Then, growth dynamics of hypoxia/reoxygenation- (H/R-) exposed hepatocytes and liver injury of HIRI-like mice were delineated after the alteration of the PIAS1 expression. We validated that PIAS1 downregulation occurred in H/R-exposed hepatocytes and HIRI-like mice, while the expression of NFATc1, HDAC1, and IRF-1 and phosphorylation levels of p38 were increased. PIAS1 inactivated p38 MAPK signaling by inhibiting HDAC1-mediated IRF-1 through NFATc1 SUMOylation, thereby repressing the inflammatory response and apoptosis of hepatocytes *in vitro*, and alleviated liver injury *in vivo*. Collectively, the NFATc1/HDAC1/IRF-1/p38 MAPK signaling axis is highlighted as a promising therapeutic target for potentiating hepatoprotective effects of PIAS1 against HIRI.

## 1. Introduction

Hepatic ischemia-reperfusion injury (HIRI) is a common complication that may occur in various clinical situations, such as liver transplantation, liver resection, trauma, and vascular surgery [1, 2]. Moreover, HIRI is one of the leading causes for postsurgery hepatic dysfunction and failure [3]. As knowledge accumulates on the molecular mechanisms underlying inflammation and apoptosis in the liver, the anticipated development and assessment of molecular targets may offer a promising perspective for HIRI [3–5].

Protein inhibitor of activated STAT1 (PIAS1), as a small ubiquitin-like modifier (SUMO) E3 ligase, was identified as a crucial player both *in vivo* and *in vitro* in numerous biological processes, such as inflammatory responses [6]. More specifically, a prior study has demonstrated the potential mechanistic action of PIAS1 as a protector against myocardial IRI by mediating PPAR*γ* SUMOylation [7]. It should be noted that PIAS1 was a negative regulator of the transactivation activity of the isoforms of nuclear factor of activated T cells 1 (NFATc1) through SUMOylation [8]. NFATc1, also known as NFAT2, is a crucial member of the transcription factor NFAT family which is indispensable in the immune system and plays an important role in liver injury repair [9, 10]. It has been reported that NFATc1 serves as a regulator of the transcriptional activity of histone deacetylase 1 (HDAC1) in its management of glioblastoma [11]. In addition, previous studies have confirmed that NFATc1 can regulate the expression of IL-2 and IL-10 in lymphocytes by interacting with HDAC1 [12, 13]. HDAC1 is responsible for many processes including cell cycle, DNA repair, and apoptosis by deacetylating histones and nonhistone proteins [14]. The inhibition of HDAC1 was confirmed to delay the progression of IRI [15]. In addition, recent data unraveled that HDAC2-mediated histone modification played a role in the development of IRI by regulating interferon regulatory factor 1 (IRF-1) in mice [16]. Furthermore, IRF-1 was verified as a vital player in HIRI by promoting autophagic cell death through the p38 mitogen-activated protein kinase (MAPK) pathway [17]. Meanwhile, the activation of p38 MAPK signaling was confirmed to exert protective effects against apoptosis and inflammation during HIRI [18].

Accordingly, the above reports suggested a potential mechanism of PIAS1 on HIRI treatment involving the NFATc1/HDAC1/IRF-1/p38 MAPK signaling axis. This study was designed to investigate the interactions among them and thus to provide a novel target for alleviating HIRI.

## 2. Materials and Methods

### 2.1. Ethics Statement

The current study was performed with the approval of the Institutional Review Board of the Second Xiangya Hospital of Central South University. All animal experiments were approved by the Animal Care and Use Committee of the Second Xiangya Hospital of Central South University and performed in strict accordance with *Guide for the Care and Use of Laboratory Animals*.

### 2.2. In Silico Analysis

Mouse HIRI-related microarray GSE10657 was obtained from the Gene Expression Omnibus (GEO) database. The microarray contains 6 sham-operated samples (sham) and 24 HIRI samples, which was annotated with the platform file GPL1261-56135. The differential analysis of HIRI-related genes was conducted using the “limma” package of *R* language, to single out differentially expressed genes (DEGs).

The correlation between DEGs and HIRI was analyzed using Phenolyzer. The protein-protein interaction (PPI) relationship network encoded by genes was analyzed by STRING (version: 11.0) with the threshold of interaction score≧0.15 and max interactors = 300.

Reperfusion injury-related genes were searched in the CTD database, and the top 1000 genes ranked by the inference score were screened out. Then, jvenn tool was employed to determine candidate genes by taking an intersection between the interaction genes and HIRI-related genes. KEGG enrichment analysis of the candidate genes was conducted using the “clusterProfiler” package of *R* language. Pearson correlation between candidate genes was conducted using Chipbase (version = 2.0) based on GETX_liver tissue expression data.

### 2.3. Cell Culture, Transduction, and Modeling

The mouse normal liver cell line AML12 was purchased from Shanghai Mingjin Biology Co., Ltd. (Shanghai, China) and cultured in DMEM/F-12 (D9785, Sigma-Aldrich, St. Louis, MO) supplemented with 10% FBS+100 U/mL penicillin and 100 *μ*g/mL streptomycin in an incubator of 5% CO_2_ at 37°C. After adherent growth, cells were detached with 0.25% trypsin (Hyclone, South Logan, UT).

AML12 cells were seeded in a 96-well plate at a density of 1 × 10^5^ cells per well and routinely cultured for 24 h. Cells were transduced with overexpression-negative control (oe-NC), oe-PIAS1, oe-NFATc1, short-hairpin RNA (sh)-NC, sh-NFATc1, sh-HDAC1#1, sh-HDAC1#2, oe-HDAC1, sh-IRF-1#1, sh-IRF-1#2, and oe-IRF-1, alone or in combination as the instructions of Lipofectamine 2000 (Invitrogen, Carlsbad, CA) described. The transduction efficiency was determined by reverse transcription-quantitative polymerase chain reaction (RT-qPCR).

The gene-overexpressing plasmid pCMV6-AC-GFP and gene-silencing plasmid pGPU6/Neo were purchased from Fenghui Biotechnology Co., Ltd. (FH1215, Hunan, China) and Shanghai GenePharma Co, Ltd. (Shanghai, China), respectively. SB203580 (S1076) (0.5 *μ*M, purchased from Selleck Chemicals, Houston, TX) was added to the medium to inhibit the activation of p38 MAPK.

The hypoxia/reperfusion (H/R) was induced in AML12 cells as described [19]. Cells were exposed to hypoxia (1% O_2_, 5% CO_2_, 94% N_2_) for 60 min and then returned to normal air conditions (95% air, 5% CO_2_).

### 2.4. Immunocytochemical Staining

The transduced cells were fixed in 4% paraformaldehyde and soaked in 0.3% Triton-X100. Cells were incubated with primary antibody rabbit anti-p-p38 (1: 1600, #4511, CST, Danvers, MA) overnight at 4°C. HRP-conjugated goat anti-rabbit IgG (H + L) secondary antibody DyLight-488 (2 *μ*g/mL, #35552, Thermo Fisher Scientific) was used on the second day. The DAB-stained sections were observed with a light microscope (Leica, Wetzlar, Germany). Five high-powered fields were randomly selected in each section with 100 cells counted in each field.

### 2.5. Coimmunoprecipitation (Co-IP)

Cells were dissolved in IP buffer for 30 min, and then the cell lysate was ultrasonically centrifuged at 20000 × g and 4°C for 10 min. The supernatant was incubated with antibody and protein A/G beads at 4°C and eluted in 1% SDS solution. The antibodies used in Western blot analysis included anti-rabbit PIAS1 (2 *μ*g, 23395-1-AP, Thermo Fisher Scientific), NFATc1 (1: 50, #8032S, CST), GAPDH (1: 60, ab181602, Abcam, Cambridge, UK), IgG (ab210935, Abcam), anti-Flag (F3165, Sigma-Aldrich), anti-HA (MMS-101P, Covance, Princeton, NJ), and anti-SUMO1 (ab32058, Abcam).

### 2.6. Western Blot Analysis

The total protein was extracted using a radioimmunoprecipitation assay (RIPA) kit (R0010, Solarbio Technology Co., Ltd., Beijing, China) containing 1% protease inhibitor and 1% phosphorylase inhibitor. The protein concentration was determined using a BCA protein assay kit. Then, 40 *μ*g of each sample was loaded, separated by 10% SDS-PAGE, and transferred onto PVDF membrane (Millipore, Billerica, MA). After being blocked with TBST solution containing 5% BSA at room temperature, blots were probed with the diluted primary antibodies overnight at 4°C and then with secondary antibodies at room temperature. The band was developed using the electrochemiluminescence (ECL) method, and images were captured on the Image Quant LAS 4000C gel imager (General Electric Company, Boston, MA). The antibodies used are as follows: rabbit anti PIAS1 (1: 2000, 23395-1-AP, Proteintech), NFATc1 (1: 1000, #8032S, CST), HDAC1 (1 *μ*g/mL, PA1-860, Thermo Fisher Scientific), IRF-1 (1: 1000, #8478S, CST), tumor necrosis factor alpha (TNF-*α*, 1: 1000, # 11948S, CST), IL-1*β* (1: 1000, ab254360, Abcam), IL-6 (1: 1000, P620, Thermo Fisher Scientific), Bcl2 (1: 2000, ab196495, Abcam), Bax (1: 2000, ab182733, Abcam), cleaved caspase-3 (1: 1000, #9664, CST), p38 (1: 1000, #8690S, CST), phosphorylated-p38 (p-p38, 1: 1000, #4511S, CST), JNK (1: 1000, ab179461, Abcam), p-JNK (1: 1000, ab124956, Abcam), ERK1/2 (1: 10000, ab184699, Abcam), p-ERK1/2 (1: 1000, ab278538, Abcam), GAPDH (1: 2500, ab9485, Abcam), and goat anti-rabbit secondary antibody (ab97051, 1: 2000, Abcam).

### 2.7. RT-qPCR

The total RNA was extracted using TRIzol (Invitrogen). The obtained total RNA was reverse transcribed into cDNA using the High-Capacity cDNA Reverse Transcription Kit (4368813, Thermo Fisher Scientific). RT-PCR experiments were performed using a SYBR®Premix Ex Taq™ (Tli RNaseH Plus) kit (RR820A, TaKaRa, Tokyo, Japan) on ABI7500 quantitative PCR machine (Thermo Fisher Scientific). The final data, as normalized to *β*-actin, were analyzed using the 2^-*ΔΔ*Ct^ method. The primers were designed and synthesized by Invitrogen. The primers used are shown in SUPPLEMENTARY Table 1.

### 2.8. Dual Luciferase Reporter Gene Assay

The 3′-UTR sequence containing the predicted binding site was inserted into the pGL3-basic vector (Promega, Madison, WI) at the XbaI restriction site of the luciferase gene downstream to generate the Firefly/Renilla luciferase reporter vector pGL3-basic-HDAC1-3′-UTR-wild type (WT, 5′-TTTTTCCTCA-3′), pGL3-basic-IRF-1-3′-UTR-WT (5′-TTTTACAGATGAGGAGAAACT-3′). The mutants are pGL3-basic-HDAC1-3′-UTR-MUT (5′-AAAAAGGAGT-3′) and pGL3-basic-IRF-1-3′-UTR-MUT (5′-AAAATGTCTACTCCTCTTTGA-3′).

HEK-293 T cells were cotransduced for 24 h with the following combinations using lipofectamine 2000: oe-NC/oe-NFATc1 + pGL3-basic-HDAC1-3′-UTR-WT, oe-NC/oe-NFATc1 + pGL3-basic-HDAC1-1-3′-UTR-MUT, and oe-NC/oe-HDAC1 + pGL3-basic-IRF-1-3′-UTR-WT, except for oe-NC/oe-HDAC1 + pGL3-basic-IRF-1-3′-UTR-MUT, and other groups were all cotransduced with 10 ng pRL-TK Renilla luciferase. The relative luciferase activity was measured using the dual luciferase reporter gene assay system (E1910, Promega), and the Renilla luciferase activity was used for normalization.

### 2.9. HIRI Mouse Model

Forty C57BL/6 male mice (aged 8-12 weeks, weight 20-26 g, 8 mice per group) were kept in separate cages with the environmental temperature at 22 ± 1°C and the light/dark cycle of 12 h/12 h, available to free drinking water and food. After one week of acclimation, the mice were randomly divided into 5 groups (8 in each group): Sham (sham operation), HIRI + oe-NC (HIRI modeling and injection of oe-NC adenovirus), HIRI + oe-PIAS1 (HIRI modeling and injection of oe-PIAS1 adenovirus), HIRI + PBS (HIRI modeling and PBS injection), and HIRI + oe-PIAS1 + anisomycin (HIRI modeling, PBS injection, and injection of p38 MAPK activator anisomycin [10 mg/mL in DMSO, 0.2 *μ*m filtered, A5862, Sigma-Aldrich]).

Mice were injected with 50 *μ*L of adeno-associated virus (GenePharma) harboring overexpression-PIAS1 (oe-PIAS1) and negative control (oe-NC) through the tail vein with 1 × 10^11^ virus copies at a concentration of 1 × 10^9^ pfu/mL (diluted with sterile PBS).

After the injection, the HIRI model was induced (SUPPLEMENTARY Figure 1) as previously described [20]. After made a midline incision by conducting laparotomy, a nontraumatic vascular clip was placed on the blood vessel blocking the blood supply of the median and left lateral lobe of the portal vein and hepatic artery for 1 h to cause ischemia. The clip was then gently removed and reperfused for 12 h, causing about 70% of liver ischemia/reperfusion injury in mice. In the sham operation group, laparotomy and vascular separation were performed without occlusion. The liver tissues of mice were used for subsequent experiments.

### 2.10. Biochemical Detection

Serum alanine aminotransferase (ALT) and aspartate aminotransferase (AST) levels were detected using ADVIA 2400 Chemistry System (Siemens, Tarrytown, NY). The inflammatory response was measured by detecting the levels of mouse serum cytokines TNF-*α*, IL-1*β*, and IL-6 using ELISA kits: murine TNF-*α* Standard ABTS ELISA Development Kit (900-T54, PeproTech, Rocky Hill, NJ) and mouse/rat Ccl2/JE/MCP-1 Quantikine ELISA Kit (MJE00, R&D Systems, Minneapolis, MN).

### 2.11. Liver Injury Evaluation

The malondialdehyde (MDA) production [21] and myeloperoxidase (MPO) activity [22] in liver tissues were assessed as described. For MDA production detection, 200 *μ*L of liver homogenate samples were mixed with the reagents provided in the KeyGEN MDA detection kit (KGT004, KeyGEN, Nanjing, China). The optical density (OD) value at 532 nm was measured using a microplate reader. In terms of MPO activity detection, 100 mg liver tissue was homogenized and centrifuged to collect pellet, which was resuspended in buffer solution containing 43.2 mmol/l KH_2_HPO_4_, 10 mmol/l EDTA, 6.5 mmol/l Na_2_HPO_4_, and 0.5% hexadecyltrimethylammonium, followed by ultrasonication for 10 s. The supernatant was reacted with 3,3′3,5′-tetramethylbenzidine for a spectrophotometer observation to measure the OD value at 655 nm.

Liver necrosis was evaluated using the hematoxylin-eosin (H&E) staining. Liver tissue samples were fixed in 10% formalin, embedded in paraffin and sectioned (4-5 *μ*m each), and then stained with hematoxylin and eosin, and damage degree was observed under a microscope (DM5000B; Leica).

### 2.12. TUNEL Staining

TUNEL staining was conducted using an In Situ Cell Death Detection Kit (11684795910, Roche, Basel, Switzerland). Then, sections were added with proteinase K working solution and incubated in a 37°C incubator for 30 min. After being immersed in blocking solution (3% H_2_O_2_), samples were added with TUNEL test solution dropwise for incubation at 37°C in the dark for 60 min. TUNEL-positive cells were photographed using a fluorescence microscope (DMi8; Leica).

### 2.13. Immunofluorescence Staining

Paraffin-embedded liver tissues were cut into 5 *μ*m sections. Cryosections fixed with acetone were used for immunohistochemical staining of Gr-1+ and CD68+ cells in mice. Sections were incubated in 5% goat serum for 1 h to prevent nonspecific antibody binding. Then, sections were incubated at 4°C overnight with the corresponding primary antibodies: mouse anti-CD68 (1: 200, ab201845, Abcam) and rabbit anti-Gr-1 (1 *μ*g/mL, PA1-511A, Thermo Fisher Scientific), followed by secondary antibody incubation for 1 h at room temperature. Secondary antibodies included goat anti-mouse IgG H&L (Alexa Fluor® 488) (1: 200, ab150113, Abcam) and goat anti-rabbit IgG (H + L) secondary antibody DyLight-488 (2 *μ*g/mL, #35552, Thermo Fisher Scientific). The nucleus was stained with DAPI (1/10000). Images were obtained using a fluorescence microscope (OLYMPUS DX51, Tokyo, Japan) and DP2-BSW software (version 2.2, Tokyo, Japan). Images were analyzed using Image Pro Plus (version 6.0, Media Cybernetics, Rockville, MD).

### 2.14. Statistical Analysis

The SPSS 21.0 statistical software (IBM Corp, Armonk, NY) was used for statistical analysis. Measurement data were expressed as mean ± standard deviation. Comparisons between two groups were analyzed by unpaired *t*-test. Comparisons among multiple groups were performed using one-way analysis of variance (ANOVA) with Tukey's post hoc test. A value of *p* < 0.05 was considered statistically significant.

## 3. Results

### 3.1. Bioinformatics Prediction for Potential Mechanism of PIAS1 in HIRI Pathogenesis

**Figure 1:**
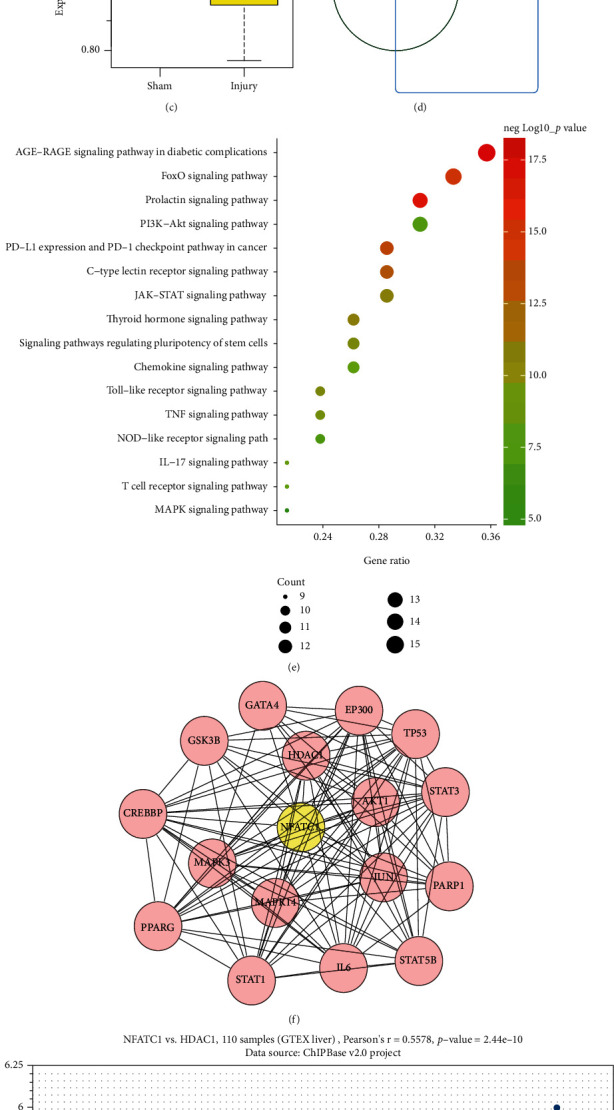
Bioinformatics analysis of the potential mechanism involved in the pathogenesis of HIRI. (a) The heat map of the top 20 DEGs with the smallest *p* value in the microarray GSE10657, the color scale from green to red indicated the gene expression value from low to high. (b) The correlation between DEGs and ischemia reperfusion injury, the X-ray indicated the correlation score. (c) The expression of PIAS1 in HIRI (*n* = 24) and sham (*n* = 6). (d) The Venn diagram of interaction factor obtained from STRING and reperfusion injury-related genes obtained from the CTD database. (e) KEGG enrichment analysis of the candidate genes. (f) The interaction network between NFATc1 and other candidate genes. (g) The interaction between NFATc1 and HDAC1 in liver tissue GTEX liver (*n* = 110) (Pearson's *r* = 0.5578, *p* value = 2.44*e*-10).

The top 20 DEGs with the smallest *p* value was screened out by analyzing the differential expression of the mouse HIRI-related microarray GSE10657 (Figure 1(a)). The correlation between the 20 DEGs and ischemia-reperfusion injury was determined using Phenolyzer, and it was revealed that PIAS1 presented the highest correlation score with ischemia-reperfusion injury (Figure 1(b)). Also, analysis of microarray GSE10657 confirmed that PIAS1 was notably lowly expressed in HIRI (Figure 1(c)). Therefore, PIAS1 was selected as the target gene for follow-up research.

In order to further predict the downstream regulatory factors of PIAS1, 300 interaction factors of PIAS1 were obtained through STRING, and an intersection between the interaction factors and ischemia-reperfusion injury-related genes was taken using the jvenn tool to obtain 43 candidate genes (Figure 1(e)). Based on the results of KEGG enrichment analysis, the 43 candidate genes were mainly involved in the AGE-RAGE signaling pathway, FoxO signaling pathway, and MAPK signaling pathway (Figure 1(e)), among which 9 genes including AKT1, CHUK, JUN, MAPK14, MAPK3, NFATc1, NFKB1, RELA, and TP53 participated in the MAPK signaling pathway. We used STRING tool to analyze the interaction between NFATc1 and other candidate genes, and it was found that NFATc1 and HDAC1 are positively correlated in liver tissue (Pearson′s *r* = 0.5578, *p* value = 2.44*e*-10) (Figures 1(f) and 1(g)).

Based on the above findings, it was inferred that the SUMO E3 ligase PIAS1 may be a key gene involved in the pathogenesis of HIRI by regulating NFATc1, HDAC1, and MAPK signaling pathway.

### 3.2. PIAS1 Inhibits Inflammatory Response and Apoptosis of Hepatocytes through NFATc1 SUMOylation

**Figure 2:**
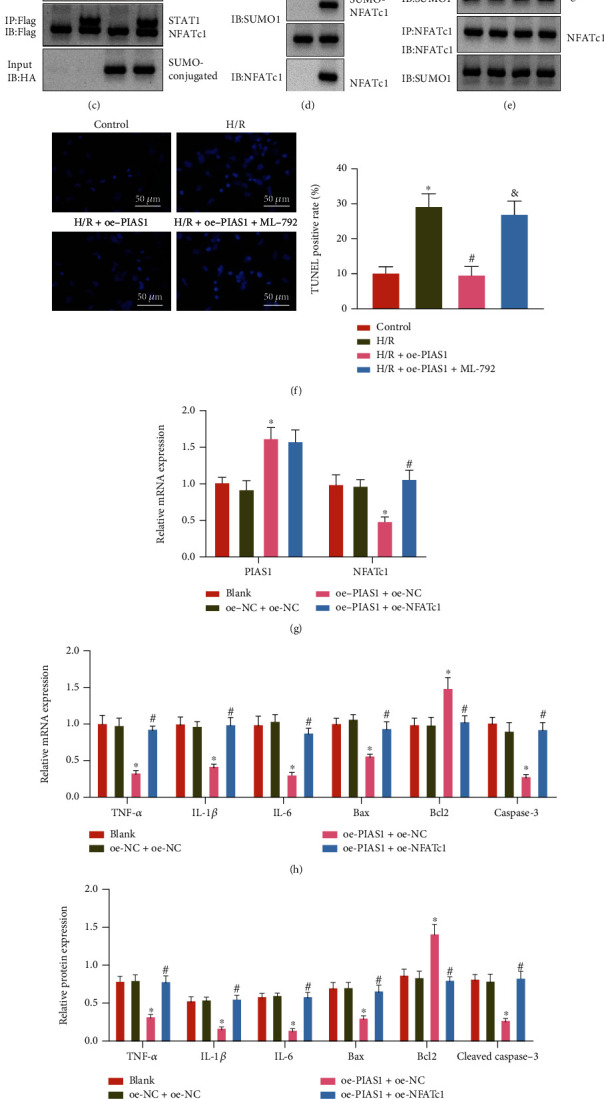
The effect of PIAS1 on NFATc1 SUMOylation and the inflammatory response and apoptosis of hepatocytes. (a) The mRNA expression of PIAS1 and NFATc1 in the liver tissues of sham-operated and HIRI mice determined by RT-qPCR (^∗^*p* < 0.05). (b) The protein expression of PIAS1 and NFATc1 in the liver tissues of sham-operated and HIRI mice determined by western blot analysis (^∗^*p* < 0.05). (c) The SUMOylation of NFATc1 in 293 T cells transduced with Flag-NFATc1 and HA-SUMO1 detected by Co-IP assay. (d) The SUMOylation of endogenous NFATc1 in AML12 cells detected by Co-IP assay. (e) The SUMOylation of NFATc1 in H/R-exposed AML12 cells treated with oe-PIAS1 or combined with ML-792 detected by Co-IP assay. (f) Apoptosis of H/R-exposed AML12 cells detected by TUNEL staining (^∗^*p* < 0.05 vs. control, #*p* < 0.05 vs. H/R, &*p* < 0.05 vs. H/R + oe-PIAS1). (g) RT-qPCR detection of PIAS1 and NFATc1 expression in H/R-exposed AML12 cells treated with oe-PIAS1 or combined with oe-NFATc1. (h) The mRNA expression of TNF-*α*, IL-1*β*, IL-6, Bax, Bcl2, and caspase-3 in H/R-exposed AML12 cells treated with oe-PIAS1 or combined with oe-NFATc1 measured by RT-qPCR. (i) The protein expression of TNF-*α*, IL-1*β*, IL-6, Bax, Bcl2, and cleaved caspase-3 in H/R-exposed AML12 cells treated with oe-PIAS1 or combined with oe-NFATc1 measured by Western blot analysis. (j) Apoptosis of H/R-exposed AML12 cells following treatment with oe-PIAS1 or combined with oe-NFATc1 detected by TUNEL staining. In (g)–(j), ^∗^*p* < 0.05 vs. oe-NC + sh-NC, #*p* < 0.05 vs. oe-PIAS1 + oe-NC. The cell experiment was repeated 3 times.

We first went to validate the expression of PIAS1 and NFATc1 in the context of HIRI by RT-qPCR and western blot analysis. It was uncovered that the expression of PIAS1 was reduced, and the expression of NFATc1 was notably increased in the liver tissues of HIRI mice (Figures 2(a) and 2(b), SUPPLEMENTARY Figure 2A).

Next, it was observed whether NFATc1 is the SUMOylation target of PIAS1 in HIRI. The SUMOylation of NFATc1 in 293 T cells transduced with Flag-NFATc1 or HA-SUMO1 was determined by conducting Co-IP experiments, and the SUMO1-NFATc1 conjugate band was observed at -152 kDa (Figure 2(c)). In addition, the SUMOylation of endogenous NFATc1 in hepatocytes was also identified by conducting Co-IP experiments using the anti-NFATc1 antibody (Figure 2(d)).

The association of PIAS1 with the SUMOylation of NFATc1 was further illuminated in H/R-exposed hepatocytes. Co-IP assay suggested that the SUMOylation of NFATc1 was much lowered in H/R-exposed hepatocytes. The PIAS1 restoration enhanced the SUMOylation of NFATc1, while it was decreased following further treatment of SUMO inhibitor ML-792 (Figure 2(e)). The TUNEL staining indicated enhanced apoptosis of H/R-exposed hepatocytes. Besides, the PIAS1 overexpression repressed apoptosis of H/R-exposed hepatocytes, which was increased in the presence of additional SUMO inhibitor ML-792 treatment (Figure 2(f)).

Furthermore, the PIAS1 expression was elevated, and the NFATc1 expression was reduced in response to oe-PIAS1 + oe-NC transduction, while the NFATc1 levels were elevated in response to oe-PIAS1 + oe-NFATc1 compared with oe-PIAS1 alone (Figure 2(g)).

In addition, the restored expression of PIAS1 resulted in repressed expression of TNF-*α*, IL-1*β*, IL-6, Bax, and caspase-3 mRNA (cleaved caspase-3 protein) as well as cell apoptosis, while the expression of Bcl2 was increased in H/R-exposed hepatocytes. Relative to oe-PIAS1 alone, simultaneous restoration of PIAS1 and NFATc1 led to the elevated expression of TNF-*α*, IL-1*β*, IL-6, Bax, and caspase-3 mRNA (cleaved caspase-3 protein) as well as cell apoptosis was increased, while the expression of Bcl2 was decreased in H/R-exposed hepatocytes (Figures 2(h)–2(j), SUPPLEMENTARY Figure 2B).

Taken together, PIAS1 reduced hepatocyte inflammatory response and apoptosis through the SUMOylation of NFATc1.

### 3.3. NFATc1 Induces Inflammatory Response and Apoptosis in Hepatocytes by Enhancing HDAC1 Transcriptional Activity

**Figure 3:**
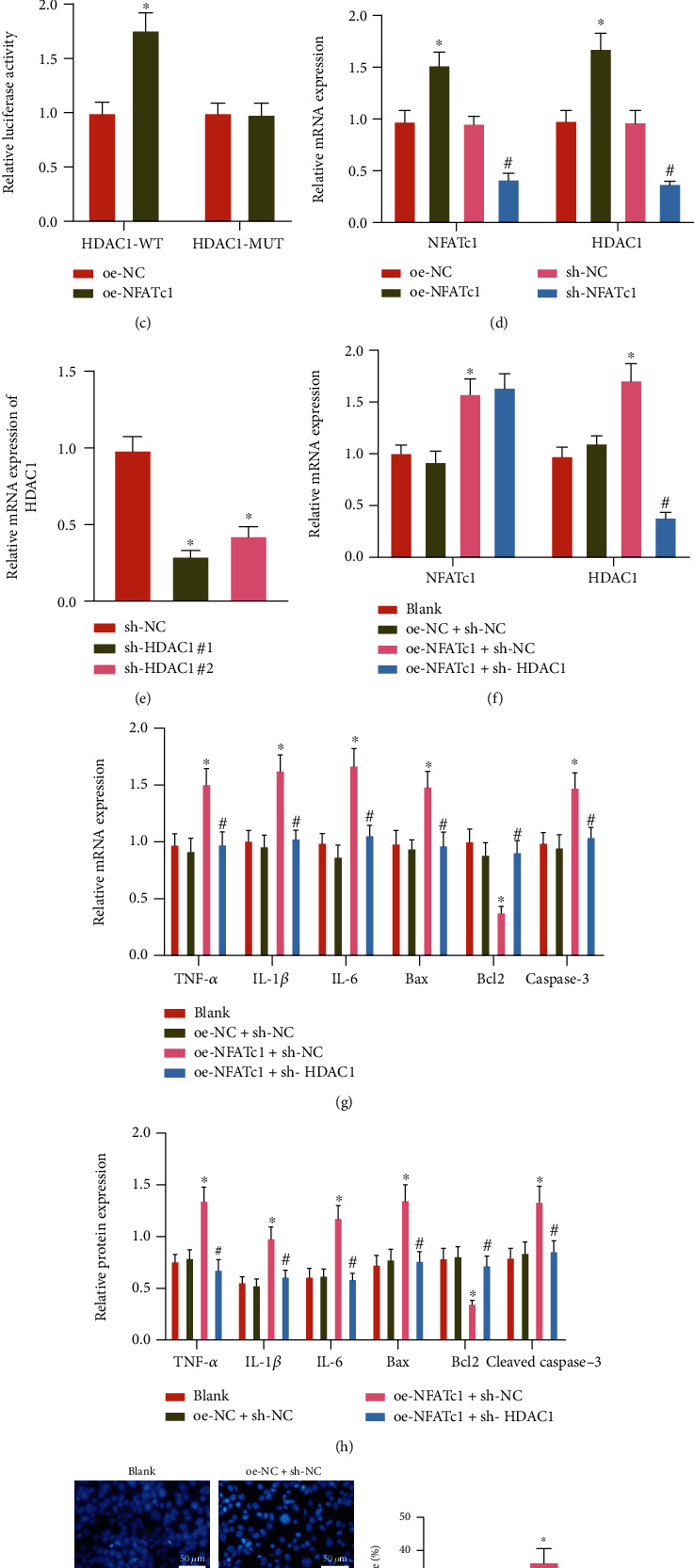
The effect of NFATc1 on HDAC1 expression and inflammatory response and apoptosis in H/R-exposed hepatocytes. (a) The expression of HDAC1 in the liver tissues of sham-operated and HIRI mice determined by RT-qPCR (^∗^*p* < 0.05). (b) The protein expression of HDAC1 in the liver tissues of sham-operated and HIRI mice determined by western blot analysis (^∗^*p* < 0.05). (c) The binding relationship between NFATc1 and HDAC1 in HEK-293 T cells transduced with oe-NFATc1 verified by dual luciferase reporter gene assay (^∗^*p* < 0.05). (d) The expression of HDAC1 in AML12 cells transduced with oe-NFATc1 or sh-NFATc1 determined by RT-qPCR (^∗^*p* < 0.05 vs. oe-NC, #*p* < 0.05 vs. sh-NC). (e) The silencing efficiency of HDAC1 in AML12 cells determined by RT-qPCR (^∗^*p* < 0.05*vs.* sh-NC). (f) RT-qPCR detection of NFATc1 and HDAC1 expression in AML12 cells transduced with oe-NFATc1 or combined with sh-HDAC1. (g) The mRNA expression of TNF-*α*, IL-1*β*, IL-6, Bax, Bcl2, and caspase-3 in AML12 cells transduced with oe-NFATc1 or combined with sh-HDAC1 determined by RT-qPCR. (h) The protein expression of TNF-*α*, IL-1*β*, IL-6, Bax, Bcl2. and cleaved caspase-3 in AML12 cells transduced with oe-NFATc1 or combined with sh-HDAC1 determined by western blot analysis. (i) AML12 cell apoptosis following transduction with oe-NFATc1 or combined with sh-HDAC1 detected by TUNEL staining. In (f)–(i), ^∗^*p* < 0.05 vs. oe-NC + sh-NC, #*p* < 0.05 vs. oe-NFATc1 + sh-NC. All cell experiments were repeated 3 times.

The effect of HDAC1 in the hepatocytes after different treatments was further assessed. It was found that the expression of HDAC1 in the liver tissues of HIRI mice was upregulated (Figures 3(a) and 3(b), SUPPLEMENTARY Figure 2C). In addition, compared with HDAC1-WT alone, the luciferase activity in the presence of oe-NFATc1 + HDAC1-WT was promoted, while there was no significant difference in luciferase activity of cells cotransduced with HDAC1-MUT, indicating that NFATc1 can bind to the promoter region of HDAC1 (Figure 3(c)).

Besides, the oe-NFATc1 and sh-NFATc1 were found to notably increase or decrease the NFATc1 expression based on RT-qPCR detection (Figure 3(d)). Moreover, the sh-HDAC1#1 led to much lower HDAC1 levels than sh-HDAC1#2, indicating that the silencing efficiency of sh-HDAC1#1 was better (Figure 3(e)). Hence, sh-HDAC1#1 was selected for following experiments.

The levels of NFATc1 and HDAC1 expression were elevated in the oe-NFATc1 presence, while the HDAC1 expression was diminished in response to oe-NFATc1 + sh-HDAC1 as compared with oe-NFATc1 alone (Figure 3(f)).

Additionally, the restored NFATc1 expression was demonstrated to elevate the levels of TNF-*α*, IL-1*β*, IL-6, Bax, and caspase-3 mRNA (cleaved caspase-3 protein) along with cell apoptosis, while the expression of Bcl2 was decreased. Relative to oe-NFATc1 alone, the levels of TNF-*α*, IL-1*β*, IL-6, Bax and caspase-3 mRNA (cleaved caspase-3 protein), and the cell apoptosis were suppressed, accompanied by the elevated Bcl2 expression in the presence of additional sh-HDAC1 treatment in H/R-exposed hepatocytes (Figures 3(g)–3(l), SUPPLEMENTARY Figure 2D).

Together, NFATc1 can induce hepatocyte inflammatory response and apoptosis by upregulating the transcriptional activity of HDAC1.

### 3.4. HDAC1 Induces Inflammatory Response and Apoptosis of Hepatocytes through Transcriptional Activation of IRF-1

**Figure 4:**
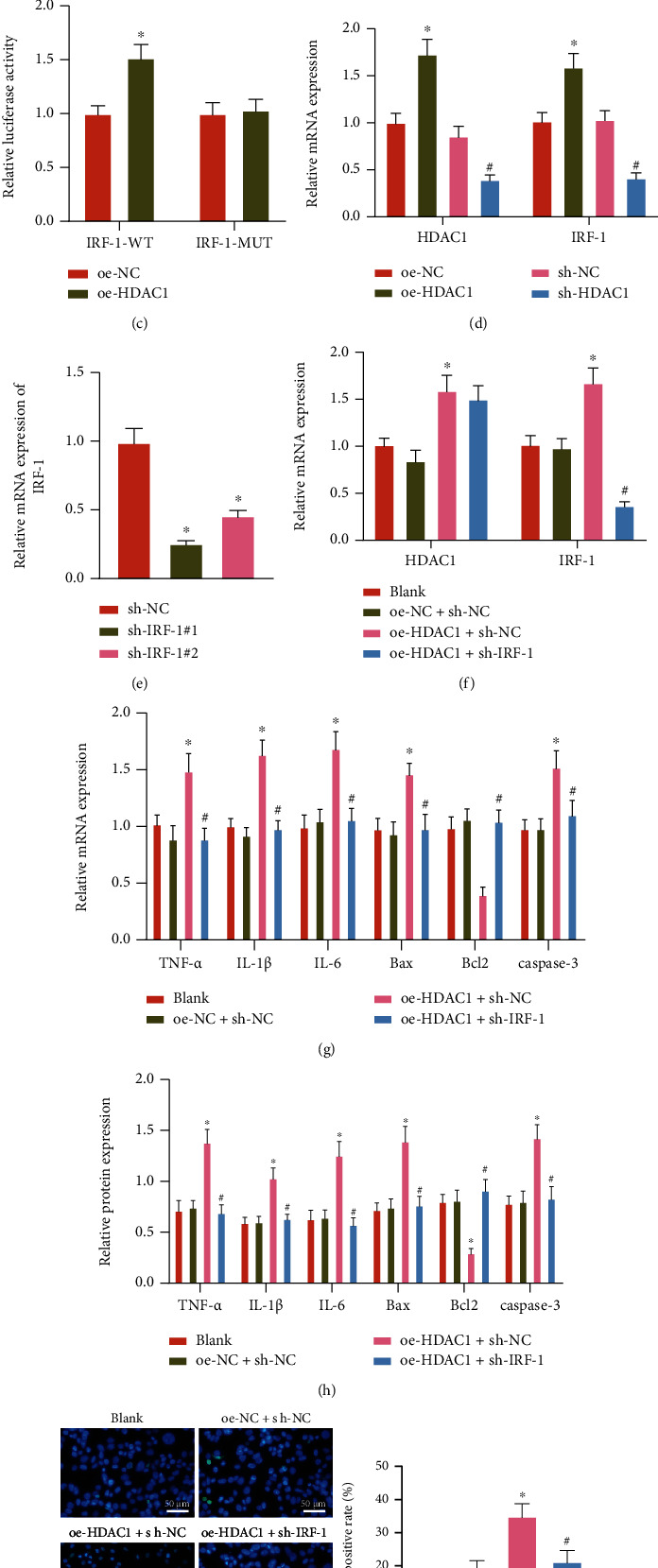
The effect of HDAC1 on IRF-1 transcription and hepatocyte inflammatory response and apoptosis in H/R-exposed hepatocytes. (a) The mRNA expression of IRF-1 in the liver tissues of sham-operated and HIRI mice determined by RT-qPCR (^∗^*p* < 0.05). (b) The protein expression of IRF-1 in the liver tissues of sham-operated and HIRI mice determined by western blot analysis (^∗^*p* < 0.05). (c) The binding between HDAC1 and IRF-1 in HEK-293 T cells transduced with oe-HDAC1 determined by dual luciferase reporter gene assay (^∗^*p* < 0.05). (d) The expression of IRF-1 in AML12 cells transduced with oe-HDAC1 or sh-HDAC1 determined by RT-qPCR (^∗^*p* < 0.05 vs. oe-NC, #*p* < 0.05 vs. sh-NC). (e) The silencing efficiency of IRF-1 in AML12 cells determined by RT-qPCR (^∗^*p* < 0.05 vs. sh-NC). (f) RT-qPCR detection of HDAC1 and IRF-1 expression in H/R-exposed AML12 cells transduced with oe-HDAC1 or combined with sh-IRF-1. (g) The mRNA expression of TNF-*α*, IL-1*β*, IL-6, Bax, Bcl2, and caspase-3 in H/R-exposed AML12 cells transduced with oe-HDAC1 or combined with sh-IRF-1 determined by RT-qPCR. (h) The protein expression of TNF-*α*, IL-1*β*, IL-6, Bax, Bcl2 ,and cleaved caspase-3 in H/R-exposed AML12 cells transduced with oe-HDAC1 or combined with sh-IRF-1 determined by western blot analysis. (i) Apoptosis of H/R-exposed AML12 cells following transduction with oe-HDAC1 or combined with sh-IRF-1 determined by TUNEL staining. In (f)–(i), ^∗^*p* < 0.05 vs. oe-NC + sh-NC, #*p* < 0.05 vs. oe-HDAC1 + sh-NC. All cell experiments were repeated 3 times.

We next moved to clarify the effect of IRF-1 in hepatocytes after different treatments. It was observed that the expression of IRF-1 in the liver tissues of HIRI mice was notably increased (Figures 4(a) and 4(b), SUPPLEMENTARY Figure 2E). Compared with cells transduced with oe-NC + IRF-1-WT, the luciferase activity of cells after the transduction of oe-HDAC1 + IRF-1-WT was increased, while there was no significant difference in luciferase activity of those with IRF-1-MUT, indicating that HDAC1 can bind to the promoter region of IRF-1 (Figure 4(c)).

Further, the expression of IRF-1 in the presence of oe-HDAC1 was obviously increased, and the expression of IRF-1 was notably decreased in the presence of sh-HDAC1 (Figure 4(d)). Moreover, the decrease of IRF-1 in cells after the transduction of sh-IRF-1#1 was much more obvious compared with those with sh-IRF-1#2 (Figure 4(e)). So, sh-IRF-1#1 was selected for follow-up experiments.

HDAC1 and IRF-1 expression was elevated in the presence of oe-PIAS1, while the results were negated in response to additional oe-NFATc1 (Figure 4(f)).

Next, it was observed in hepatocytes that the expression of TNF-*α*, IL-1*β*, IL-6, Bax, and caspase-3 mRNA (cleaved caspase-3 protein) along with cell apoptosis was increased, while the expression of Bcl2 was decreased in response to oe-HDAC1, but the results were counteracted in the presence of oe-HDAC1 + sh-IRF-1 (Figures 4(g)–4(I), SUPPLEMENTARY Figure 2F).

To sum up, HDAC1 promoted hepatocyte inflammatory response and apoptosis through transcriptional activation of IRF-1.

### 3.5. IRF-1 Promotes Inflammatory Response and Apoptosis of Hepatocytes by Activating the p38 MAPK Signaling Pathway

**Figure 5:**
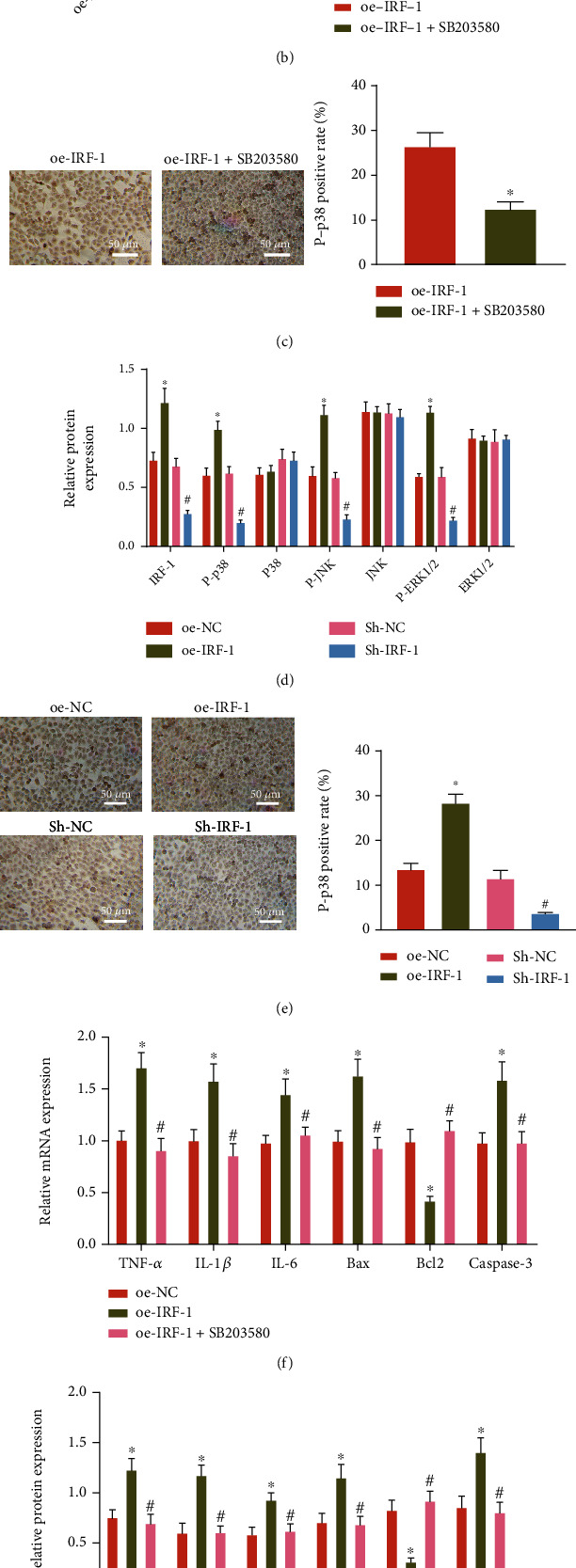
IRF-1 promoted inflammatory response and apoptosis in H/R-exposed hepatocytes by activating p38 MAPK signaling pathway. (a) The phosphorylation levels of p38, JNK, and ERK1/2 in the liver tissues of sham-operated and HIRI mice determined by Western blot analysis (^∗^*p* < 0.05). (b) The phosphorylation level of p38 in AML12 cells treated with sh-IRF-1 or combined with SB203580 (p38 MAPK inhibitor) determined by western blot analysis (^∗^*p* < 0.05). (c) The phosphorylation level of p38 in AML12 cells treated with sh-IRF-1 or combined with SB203580 determined by immunocytochemical staining (^∗^*p* < 0.05). (d) The phosphorylation levels of p38, JNK, and ERK1/2 in AML12 cells treated with sh-IRF-1 or oe-IRF-1 detected by Western blot analysis (^∗^*p* < 0.05 vs. oe-NC, #*p* < 0.05 vs. sh-NC). (e) The phosphorylation levels of p38, JNK, and ERK1/2 in AML12 cells treated with sh-IRF-1 or oe-IRF-1 detected by immunocytochemical staining (^∗^*p* < 0.05 vs. oe-NC, #*p* < 0.05 vs. sh-NC). (f) The mRNA expression of inflammatory response and apoptosis-related proteins in AML12 cells treated with oe-IRF-1 or combined with SB203580 determined by RT-qPCR. (g) The protein expression of inflammatory response and apoptosis-related proteins in AML12 cells treated with oe-IRF-1 or combined with SB203580 determined by Western blot analysis. (h) AML12 cell apoptosis following treatment with oe-IRF-1 or combined with SB203580 determined by TUNEL staining. In (f)–(h), ^∗^*p* < 0.05 vs. oe-NC, #*p* < 0.05 vs. oe-IRF-1. Cell experiments were all repeated 3 times.

First, the phosphorylation levels of p38, JNK, and ERK1/2 in hepatocytes after different treatments were determined by western blot analysis. It was demonstrated that the phosphorylation levels of p38, JNK, and ERK1/2 were notably increased in the liver tissues of HIRI mice (Figure 5(a)). In order to confirm whether the role of IRF-1 in HIRI depends on the p38 MAPK signaling pathway, hepatocytes were transduced with oe-IRF-1, the phosphorylation level of p38 before and after the addition of the p38 MAPK inhibitor SB203580 was observed, and it was uncovered that the phosphorylation level of p38 increased obviously after adding the p38 MAPK inhibitor SB203580 (Figures 5(b) and 5(c)).

According to the results of western blot analysis and immunocytochemical staining, the phosphorylation levels of p38, JNK, and ERK1/2 in cells transduced with oe-IRF-1 were notably increased. The phosphorylation levels of p38, JNK, and ERK1/2 were decreased in cells after the transduction of sh-IRF-1 (Figures 5(d) and 5(e), SUPPLEMENTARY Figure 2G). The expression of TNF-*α*, IL-1*β*, IL-6, Bax, and caspase-3 mRNA (cleaved caspase-3 protein) along with cell apoptosis was augmented while the expression of Bcl2 was downregulated in cells transduced with oe-IRF-1. Conversely, further treatment of SB203580 decreased the expression of TNF-*α*, IL-1*β*, IL-6, Bax, and caspase-3 mRNA (cleaved caspase-3 protein) along with cell apoptosis and increased the expression of Bcl2 (Figures 5(f)–5(h), SUPPLEMENTARY Figure 2H).

In short, inflammatory response and apoptosis of hepatocytes were enhanced by IRF-1 via activating the p38 MAPK signaling pathway.

### 3.6. PIAS1 Overexpression Plays a Protective Role in HIRI Mice by Inhibiting the Activation of NFATc1/HDAC1/IRF-1/p38 MAPK Pathway

**Figure 6:**
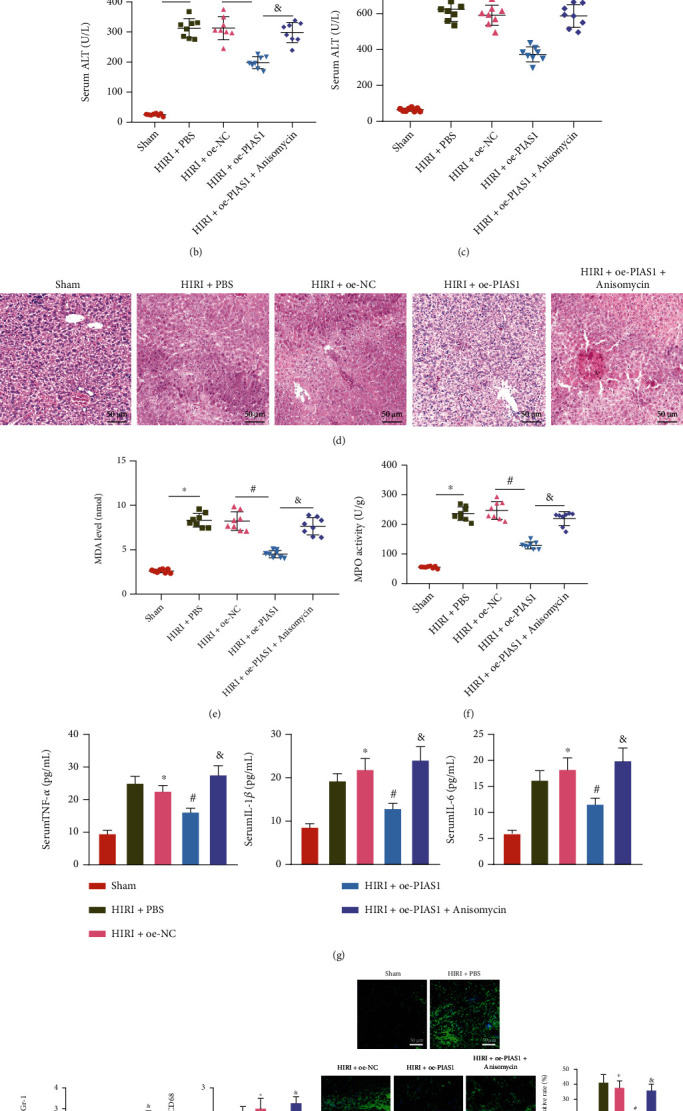
Overexpression of PIAS1 inactivated the NFATc1/HDAC1/IRF-1/p38 MAPK pathway to alleviate HIRI in mice. HIRI mice were treated with oe-PIAS1 or combined with anisomycin (p38 MAPK activator). (a) The protein expression of PIAS1, NFATc1, HDAC1, IRF-1, and phosphorylation levels of p38 in the liver tissue of mice determined by western blot analysis. (b) The serum ALT activity in mice determined by ELISA. (c) The serum AST activity in mice determined by ELISA. (d) The degree of liver injury in mice determined by H&E staining. (e) The degree of liver injury in mice determined by MDA detection. (f) The neutrophil activity in the liver tissue of mice determined by MPO detection. (g) The expression of TNF-*α*, IL-1*β*, and IL-6 levels in the liver tissue of mice determined by ELISA. (h) The levels of Gr-1 and CD68 in liver tissues of mice determined by immunofluorescence staining. (i) Hepatocyte apoptosis in liver tissues of mice determined by TUNEL assay. *n* = 8, ^∗^*p* < 0.05 vs. sham-operated mice, #*p* < 0.05 vs. HIRI + oe-NC, &*p* < 0.05 vs. HIRI + oe-PIAS1.

The *in vitro* findings on the molecular mechanism of PIAS1 were further substantiated in a HIRI mouse model. We also validated downregulated PIAS1 expression and upregulated expression of NFATc1, HDAC1, and IRF-1 and phosphorylation levels of p38 in the liver tissue of mice following HIRI versus mice receiving sham operation. Moreover, the expression of NFATc1, HDAC1, and IRF-1 and phosphorylation levels of p38 was decreased in the liver tissues of mice when the PIAS1 expression was restored in HIRI mice. Besides, additional anisomycin treatment in PIAS1-overexpressing HIRI mice, the phosphorylation levels of p38 were elevated in the liver tissue of the mice (Figure 6(a), SUPPLEMENTARY Figure 2(I)).

The activities of AST and ALT were promoted, liver injury was worsened, and the necrotic area, eosinophils, the MDA level, and MPO activity were increased in HIRI mice versus mice receiving sham operation. The restored PIAS1 expression suppressed activities of AST and ALT, alleviated liver injury, and diminished MDA level and MPO activity in HIRI mice. Further administration of anisomycin negated the effect of PIAS1 and aggravated liver injury (Figures 6(b)–6(f)).

According to the results of ELISA, immunofluorescence staining, and TUNEL staining, the serum level of TNF-*α*, IL-1*β*, and IL-6, the expression of neutrophil marker Gr-1 and macrophage marker CD68, and hepatocyte apoptosis were all promoted in the liver tissues of the HIRI mice, as compared with mice receiving sham operation. Meanwhile, the PIAS1 overexpression in HIRI mice repressed the levels of TNF-*α*, IL-1*β*, IL-6, Gr-1, CD68, and hepatocyte apoptosis and the effects of which was counterweighed following further treatment of anisomycin (Figures 6(g)–6(I)).

Taken together, the overexpression of PIAS1 inactivated the NFATc1/IRF-1/p38 MAPK pathway, thereby exerting a protective effect on HIRI mice.

## 4. Discussion

The obtained evidence suggested that PIAS1 reduced the inflammatory response and apoptosis of inactivating the p38 MAPK signaling by downregulating HDAC1-mediated IRF-1 through NFATc1 downregulation, thus ultimately alleviating HIRI-like symptoms.

A prior study has explored and confirmed the therapeutic effects of PIAS1 on protecting against myocardial IRI [23]. However, detailed studies about the downstream mechanism of PIAS1 in HIRI are still insufficient. The finding of our work suggested that PIAS1 reduced the inflammatory response and apoptosis of hepatocytes through the SUMOylation of NFATc1, evidenced by the increased Bcl2 expression and the decreased expression of TNF-*α*, IL-1*β*, IL-6, Bax, and caspase-3 in mice with HIRI-like symptom. SUMOylation was recognized as a posttranslational modification which is involved in various crucial cellular functions, such as cell cycle, DNA damage repair, and cell apoptosis [24]. Consistent with our finding, the negative regulation of PIAS1 on NFATc1 isoforms was also validated in a prior study [8]. Likewise, NFATc1 inhibition is responsible for the repressed inflammatory response [25]. More importantly, NFAT was illustrated to be positively correlated with inflammation and apoptosis and thus served as a crucial player for IRI [23]. Additionally, TNF-*α*, IL-1*β*, and IL-6 were well-known proinflammatory cytokines, and their downregulation is a biomarker of the relieved inflammation in HIRI [8]. Likewise, Bax, caspase-3, and Bcl2 were apoptosis-related protein markers, and the downregulation of Bax and caspase-3 and upregulation of Bcl2 are also identified as a symptom of repressed apoptosis in HIRI [26]. In a word, PIAS1 was a promising inhibitor for the inflammatory response and apoptosis of hepatocytes and thus further a vital mediator of HIRI through the SUMOylation of NFATc1.

It was then confirmed that NFATc1 elevated the expression of HDAC1 and subsequently activated the IRF-1-mediated p38 MAPK signaling, which further promoted the inflammatory response and apoptosis of hepatocytes. Similar to our finding, the positive correlation between NFAT and HDACs was also certified by a recent study and was confirmed as key promoter in the regulation of inflammation during the preservation of islet function after islet transplantation [27]. Besides, inhibition of HDAC1 exerted a neuroprotective effect against cerebral IRI [28, 29]. Furthermore, a prior study demonstrated that the increased expression of HDAC1 was partially responsible for the deterioration of mice with HIRI-like symptoms, and its repression acted as a promising therapeutic target for HIRI management [30, 31]. The positive regulation between HDAC and IRF-1 was also demonstrated in another study [32]. IRF-1, a transcriptional regulator of IFNs and IFN-inducible genes, is also a well-recognized vital player in response to inflammation [33]. Moreover, elevation of IRF-1 in hepatocytes was illustrated as a main cause of the enhanced apoptosis and thus further contributed to the pathogenesis of HIRI [34]. Besides, a prior study also identified the implication of IRF-1 in the progression of HIRI by positively mediating the p38 MAPK signaling pathway [17]. Additionally, activated p38 MAPK signaling pathway has been observed in HIRI [35]. In addition, inactivation of the p38 MAPK signaling represents a potential therapeutic target for HIRI [36].

## 5. Conclusion

**Figure 7:**
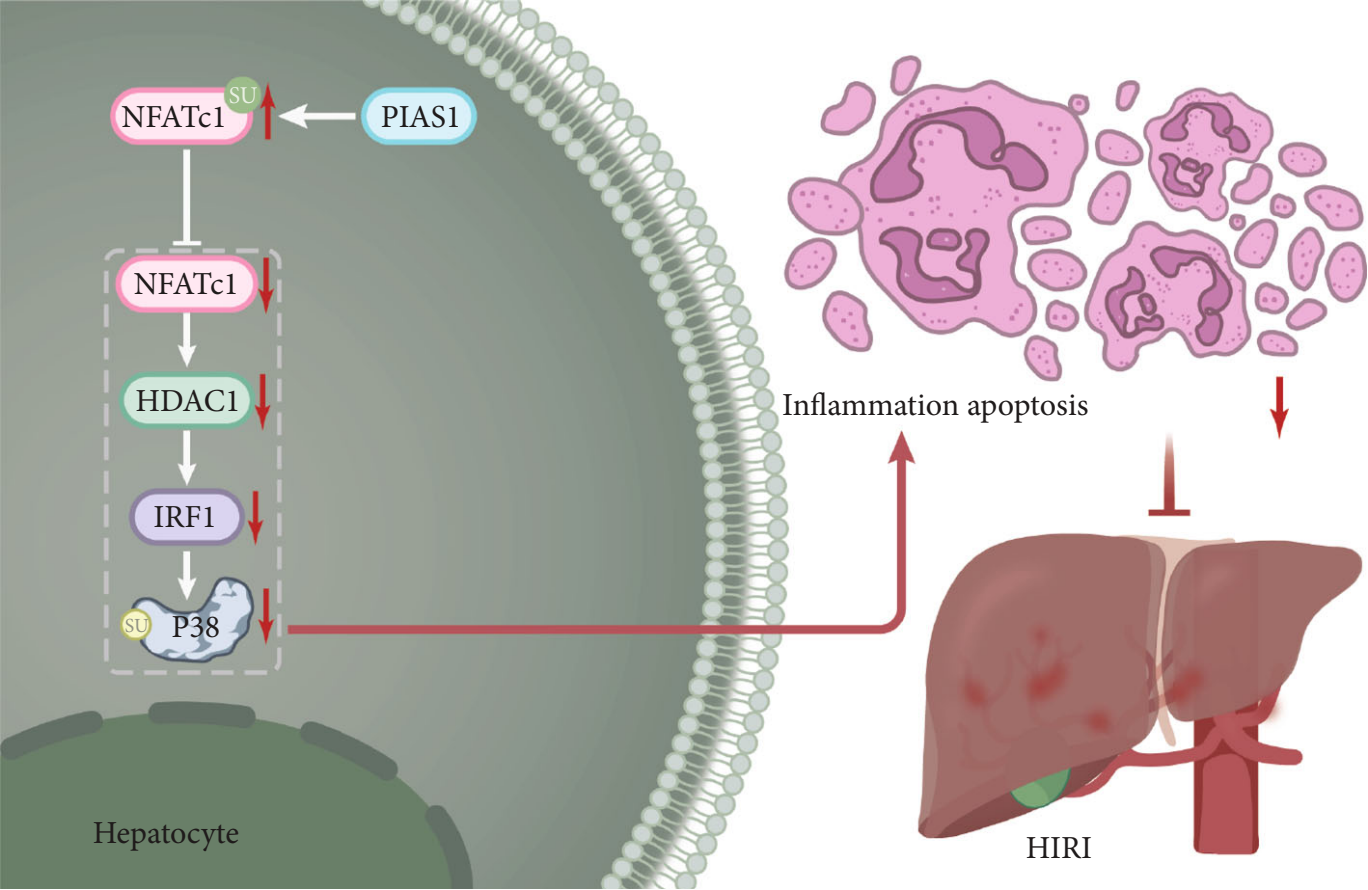
The molecular mechanism of SUMO E3 ligase PIAS1 in HIRI through regulating NFATc1/HDAC1/IRF-1/p38 MAPK signaling pathway.

In summary, SUMO E3 ligase PIAS1 alleviated HIRI by inhibiting the NFATc1/HDAC1/IRF-1/p38 MAPK pathway (Figure 7). This study provides new insights for revealing the molecular mechanism of hepatoprotective PIAS1 in HIRI. Novel therapeutics based on PIAS1 upregulation may become potential target of personalized strategy in the future for enhancing therapeutic outcomes of HIRI.

## Data Availability

The data and materials of the study can be obtained from the corresponding author upon request.

## Supplementary Materials

Supplementary Materials Supplementary Figure 1: surgical process of HIRI in mice. Supplementary Figure 2: representative western blots for quantification of the band intensities of Figures 2(b) (a), 2(i) (b), 3(b) (c), 3(h) (d), 4(b) (e), 4(h) (f), 5(d) (g), 5(g) (h), and 6(a) (i). Supplementary Table 1: primer sequences of RT-qPCR.
