# Supplementary material for: PIAS1 Alleviates Hepatic Ischemia-Reperfusion Injury in Mice through a Mechanism Involving NFATc1 SUMOylation
Source: Dis Markers. 2022 Aug 31;2022:4988539. doi: 10.1155/2022/4988539 (PMC9452975; doi:10.1155/2022/4988539)
Supplement: Supplementary Materials — Supplementary Figure 1: surgical process of HIRI in mice. Supplementary Figure 2: representative western blots for quantification of the band intensities of Figures 2(b) (a), 2(i) (b), 3(b) (c), 3(h) (d), 4(b) (e), 4(h) (f), 5(d) (g), 5(g) (h), and 6(a) (i). Supplementary Table 1: primer sequences of RT-qPCR. [file 4988539.f1.docx]

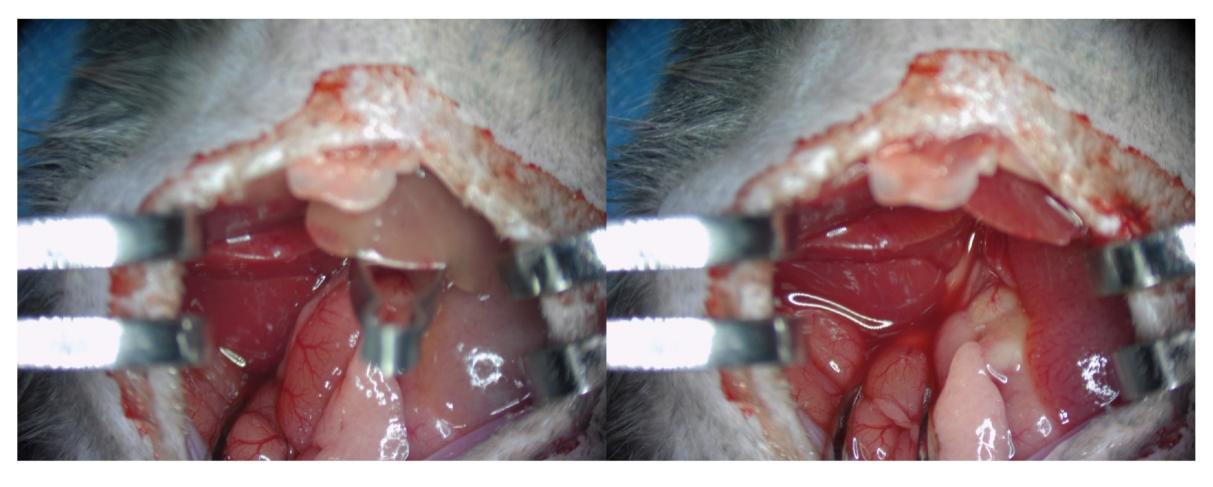


**SUPPLEMENTARY FIGURE 1** Surgical process of HIRI in mice.


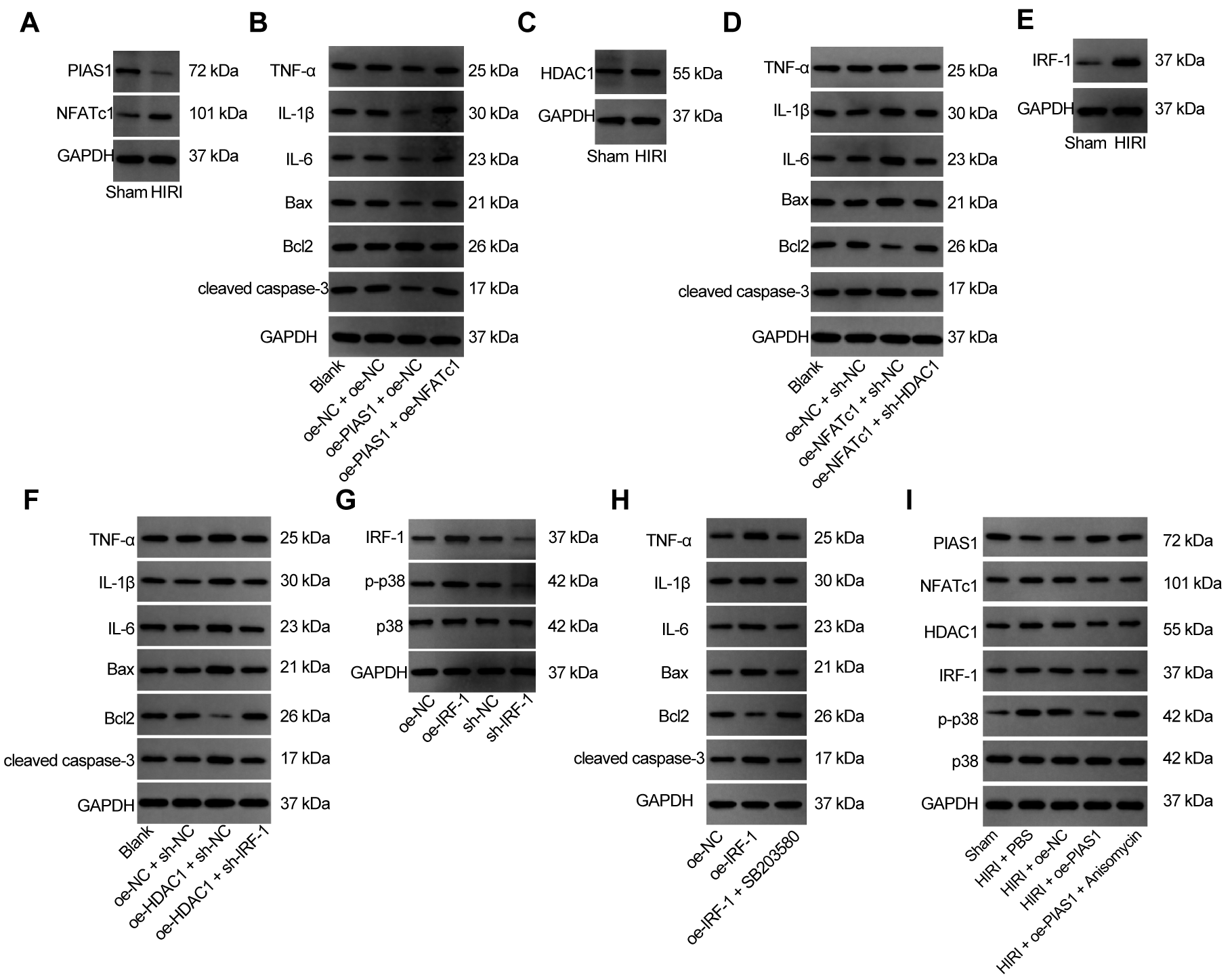


**SUPPLEMENTARY FIGURE 2** Representative Western blots for quantification of the band intensities of Figure 2B (A), 2I (B), 3B (C), 3H (D), 4B (E), 4H (F), 5D (G), 5G (H) and 6A (I).

**Supplementary Table 1 Primer sequences of RT-qPCR.**

| Genes | Primer sequence |
| --- | --- |
| PIAS1 | Forward: 5'-AAGTACTGTTGGGCTACGCT-3' |
|  | Reverse: 5'-TGAGCTGTGGAATGGTGGAT-3' |
| NFATc1 | Forward: 5'-AGATGGTGCTGTCTGGCCATAACT-3' |
|  | Reverse: 5'-TGCGGAAAGGTGGTATCTCAACAA-3' |
| HDAC1 | Forward: 5'-TGAAGCCTCACCGAATCCGCAT-3' |
|  | Reverse: 5'-TGGTCATCTCCTCAGCATTGGC-3' |
| IRF-1 | Forward: 5'-CTGGTCTTGCTGGGTACTGA-3' |
|  | Reverse: 5'-ATTCAGCCCCAAACCGTTTC-3' |
| TNF-α | Forward: 5'-GACAGTGACCTGGACTGTGG-3' |
|  | Reverse: 5'-TGAGACAGAGGCAACCTGAC-3' |
| IL-1β | Forward: 5'-GAAGAAGAGCCCATCCTCTG-3' |
|  | Reverse: 5'-TCATCTCGGAGCCTGTAGTG-3' |
| IL-6 | Forward: 5'-CTGCAAGAGACTTCCATCCAG-3' |
|  | Reverse: 5'-AGTGGTATAGACAGGTCTGTTGG-3' |
| Bax | Forward: 5'-AGACAGGGGCCTTTTTGCTAC-3' |
|  | Reverse: 5'-AATTCGCCGGAGACACTCG-3' |
| Bcl2 | Forward: 5'- AGCATGCGACCTCTGTTTGA-3' |
|  | Reverse: 5'- GCCACACGTTTCTTGGCAAT-3' |
| Caspase-3 | Forward: 5'-CTCGCTCTGGTACGGATGTG-3' |
|  | Reverse: 5'-TCCCATAAATGACCCCTTCATCA-3' |
| β-actin | Forward: 5'-GGCTGTATTCCCCTCCATCG-3' |
|  | Reverse: 5'-CCAGTTGGTAACAATGCCATGT-3' |

Note: PIAS1, protein inhibitor of activated STAT1; NFATc1, nuclear factor of activated T cells 1; HDAC1, histone deacetylase 1; IRF-1, interferon regulatory factor 1; TNF-α, tumor necrosis factor alpha; IL-1β, interleukin 1 beta; IL-6, interleukin 6; Bax, BCL2-associated X protein; Bcl2, B cell leukemia/lymphoma 2; RT-qPCR, reverse transcription quantitative polymerase chain reaction
